# What Can We Conclude from Death Registration? Improved Methods for Evaluating Completeness

**DOI:** 10.1371/journal.pmed.1000262

**Published:** 2010-04-13

**Authors:** Christopher J. L. Murray, Julie Knoll Rajaratnam, Jacob Marcus, Thomas Laakso, Alan D. Lopez

**Affiliations:** 1 Institute for Health Metrics and Evaluation, University of Washington, Seattle, Washington, United States of America; 2 School of Engineering and Applied Sciences, Harvard University, Cambridge, Massachusetts, United States of America; 3 School of Population Health, The University of Queensland, Brisbane, Australia; World Health Organization, Switzerland

## Abstract

**Background:**

One of the fundamental building blocks for determining the burden of disease in populations is to reliably measure the level and pattern of mortality by age and sex. Where well-functioning registration systems exist, this task is relatively straightforward. Results from many civil registration systems, however, remain uncertain because of a lack of confidence in the completeness of death registration. Incomplete registration systems mean not all deaths are counted, and resulting estimates of death rates for the population are then underestimated. Death distribution methods (DDMs) are a suite of demographic methods that attempt to estimate the fraction of deaths that are registered and counted by the civil registration system. Although widely applied and used, the methods have at least three types of limitations. First, a wide range of variants of these methods has been applied in practice with little scientific literature to guide their selection. Second, the methods have not been extensively validated in real population conditions where violations of the assumptions of the methods most certainly occur. Third, DDMs do not generate uncertainty intervals.

**Methods and Findings:**

In this paper, we systematically evaluate the performance of 234 variants of DDM methods in three different validation environments where we know or have strong beliefs about the true level of completeness of death registration. Using these datasets, we identify three variants of the DDMs that generally perform the best. We also find that even these improved methods yield uncertainty intervals of roughly ± one-quarter of the estimate. Finally, we demonstrate the application of the optimal variants in eight countries.

**Conclusions:**

There continues to be a role for partial vital registration data in measuring adult mortality levels and trends, but such results should only be interpreted alongside all other data sources on adult mortality and the uncertainty of the resulting levels, trends, and age-patterns of adult death considered.

*Please see later in the article for the Editors' Summary*

## Abstract 2

Julie Rajaratnam and colleagues evaluate the performance of a suite of demographic methods that estimate the fraction of deaths registered and counted by civil registration systems, and identify three variants that generally perform the best.

## Editors' Summary

**Background:**

Accurate worldwide information on the levels and patterns of mortality (deaths) is essential for planning and monitoring global public-health initiatives. The gold standard method for collecting such information is death registration. In high-income countries, death registration is effectively 100% complete, but the situation in many developing countries is very different. In most African countries, for example, less than one-quarter of deaths are officially recorded. Although other data sources such as household surveys can be used to estimate mortality levels in such countries, partial registration data could provide useful information about mortality levels in developing countries if its completeness could be evaluated. One way to do this is to use demographic methods called “death distribution methods” (DDMs). Demography is the study of the size, growth, and other characteristics of human populations; DDMs compare the age distribution of recorded deaths (the relative proportion of deaths in each age group) with the age distribution of the population in which they occurred to provide a correction factor that can be used to calculate corrected mortality levels from registered deaths. DDMs are used by the World Health Organization to monitor adult mortality in nearly 100 countries.

**Why Was This Study Done?:**

Although widely used, few studies have compared the performance of the many available DDM variants, and DDMs have not been extensively validated by testing them in populations for which the completeness of death registration is known. In addition, because DDMs are mathematical in nature, they do not provide any indication of the uncertainty associated with the correction factors they yield. This means that public-health officials using estimates of mortality levels generated from partial registration data using DDMs have no idea of the limits between which the true mortality levels of their populations lie. In this study, the researchers systematically evaluate the performance of 234 DDM variants and use the optimal variants that they identify to analyze registration completeness over time in six developing countries.

**What Did the Researchers Do and Find?:**

The researchers constructed 234 DDM variants by combining each of three general types of DDMs with 78 different “age trims”; demographers often age-trim—drop older and/or younger age groups—when using DDMs to estimate correction factors for observed death rates. The researchers then evaluated the performance of the variants in three “validation” datasets for which the completeness of death registration is known—a microsimulation of a population of 10 million people followed for 150 years, population data from US counties between 1990 and 2000, and population data from high-income OECD (Organisation for Economic Co-operation and Development) countries with populations of more than 5 million between 1950 and 2000. Detailed analyses of the performance of the DDM variants with all three datasets identified three optimal DDMs, one of each type. However, even with these optimal DDMs, the uncertainty intervals associated with estimates of relative completeness of registration were roughly +/− one-quarter of the estimate. Finally, the researchers applied their optimal DDMs to six developing countries over time. This analysis showed that death registration for adults has been relatively complete since 1970 in Mexico, for example, whereas in Tunisia, death registration has improved from nearly 50% in 1965 to complete by 1980. It also indicated that the three DDMs can give consistent results in some contexts.

**What Do These Findings Mean?:**

By using multiple validation databases, these findings identify three optimal DDMs for the estimation of completeness of death registration. The researchers recommend that analysts apply all three methods when estimating the completeness of death registration data and look at the consistency of the results produced. They warn that the level of uncertainty associated with the estimation of completeness of registration means that results yielded by DDMs should be interpreted with considerable caution. In particular, they note that although correction factors provided by DDMs may be a good way of estimating mortality levels, the uncertainty in these factors may make them unsuitable for analyzing trends over time in mortality levels. Overall, the researchers conclude that partial death registration data have a role to play in measuring adult mortality levels, provided that they are analyzed alongside other data sources.

**Additional Information:**

Please access these Web sites via the online version of this summary at http://dx.doi.org/10.1371/journal.pmed.1000262.

- This study and two related *PLoS Medicine* Research Articles—by Obermeyer et al. and by Rajaratnam et al. —are further discussed in a *PLoS Medicine* Perspective by Mathers and Boerma
- The Institute for Health Metrics and Evaluation makes available high-quality information on population health, its determinants, and the performance of health systems
- Grand Challenges in Global Health provides information on research into better ways for developing countries to measure their health status
- The World Health Organization Statistical Information System (WHOSIS) is an interactive database that brings together core health statistics for WHO member states, including information on vital registration of deaths; the WHO Health Metrics Network is a global collaboration focused on improving sources of vital statistics

## Introduction

One of the fundamental building blocks for determining the burden of disease in populations is to reliably measure the level and pattern of mortality by age and sex. Simply knowing death rates at specific ages is itself an important descriptor of the epidemiological situation in a population, given the strong age dependence of major diseases and injuries. After decades of effort and emphasis on improving survival among children, uncertainty about levels and trends in child mortality has been substantially reduced (although further improvements in knowledge are possible with better methods and wider access to survey and census data [1]). This improvement is not the case with adult mortality, despite the focus on adult health outcomes in Millennium Development Goal (MDG) 5 (reducing maternal mortality) and MDG 6 (halting and reversing the spread of HIV, tuberculosis, and malaria).

Given the importance of estimating underlying mortality rates in order to more reliably describe the burden of disease in populations, particularly for populations where the routine registration of deaths functions poorly, methods have been developed to more successfully exploit the substantial amount of information on the survival of siblings that has been collected in large-scale global survey programs [2]. For many developing countries, however, the mainstay for adult mortality measurement remains civil registration systems. Over 50 developing countries annually report death statistics to the World Health Organization (WHO) or the United Nations Statistics Division [3],[4]. Results from data collected using civil registration systems, however, remain uncertain owing to a lack of confidence in the completeness of death registration and the accuracy of reports about age at death.

Beginning in the 1960s and 1970s, methods were developed by demographers in an attempt to estimate the completeness of death reporting, either in civil registration systems or in censuses and surveys [5]–[11]. These methods, known in the literature as death distribution methods (DDMs), are effectively based on a comparison of the age distribution of recorded deaths with the age distribution of the population in which the deaths occurred. In order to satisfy the basic demographic balancing equation (namely, that population growth is a function of births, deaths, and migration), the methods are dependent on assumptions that birth and death rates are constant, that there is no net migration in the population, and that the extent of age misreporting and other errors in data collection are minimal.

These methods have been widely applied to census and vital registration data in the literature and are used for nearly 100 countries by WHO to monitor adult mortality [4],[12]–[19]. Although widely applied and used, the methods have at least three types of limitations. First, a wide range of variants of these methods has been applied in practice with little scientific literature to guide selection of these variants (we describe these possible variants in greater detail in the Methods). Second, the methods have not been extensively validated in real populations in the presence of measurement error and other violations of the assumptions. We know of only two studies evaluating the performance of these methods to date. One study [20] found large variation in results when applied to high-income countries where registration is thought to be complete. Another study [21] found the methods to be accurate when their assumptions were not violated; however, the margins of error grew quite extensive in the presence of violations. Both studies were conducted using a limited set of the possible variants of these methods and were examined in a limited range of population scenarios. Third, DDM methods are grounded in mathematical, not statistical, relationships and thus do not generate uncertainty intervals for the estimated completeness of death registration.

Here, we aim to systematically evaluate the performance of 234 variants of DDMs on three different validation datasets and, on the basis of this evaluation, to develop improvements to the application of these methods in countries with incomplete vital registration.

## Methods

### Three DDM Families

**Figure 1:**
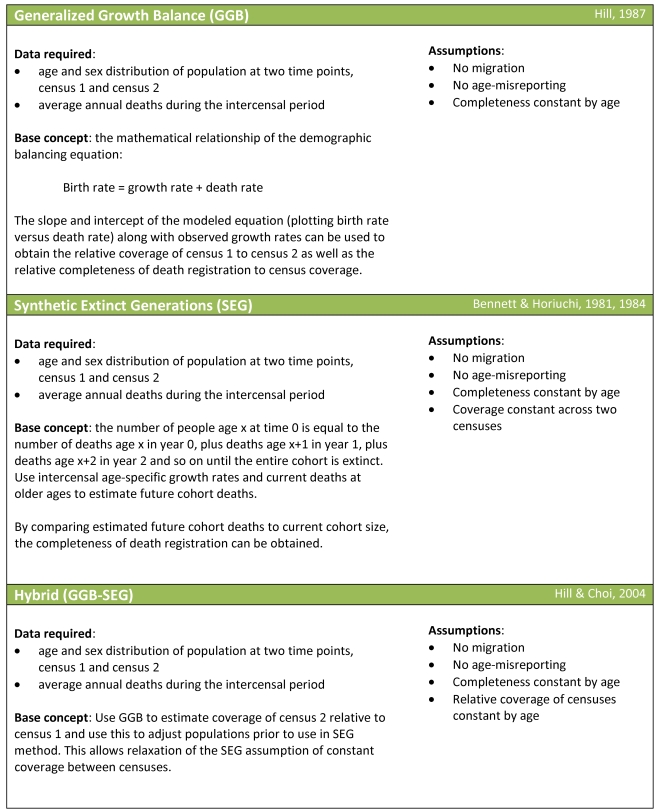
Three families of DDMs.

The three families of methods used for assessing the completeness of death registration are generalized growth balance (GGB), synthetic extinct generations (SEG), and a hybrid of the two approaches (GGBSEG) (Figure 1). Text S1 provides a brief summary of these methods and the mathematical relationships that underlie them. All that these methods require as input are age distributions of population from two censuses and the deaths registered between the censuses by age. The methods are normally applied separately to males and females. In some cases, instead of death captured by vital registration systems, deaths reported in a census in the last 12 mo have been used. All three families of methods ultimately yield a correction factor that can be multiplied by the observed adult death rates to get the corrected adult death rates (see Text S1). SEG methods yield an estimate of the completeness of death registration relative to the two censuses. GGB methods and the related GGBSEG yield an estimation of the completeness of census 2 relative to census 1 as well as the completeness of death registration relative to the censuses. In theory, GGB and GGBSEG should perform better in the presence of differential completeness of the two censuses.

On the basis of practical experience, results of applying DDMs to population and death data for all adult age groups can give findings that lack face validity. Demographers often age-trim, namely drop the older and/or younger age groups in the estimation of the correction factor for observed death rates. This practical approach has a sound theoretical basis: the effects of random fluctuations in the number of deaths or the population in some age groups, age misreporting, and migration may vary substantially at older or younger age groups. While age trimming is widely practiced, there are no published studies that systematically evaluate the performance of different age trims for the three families of DDMs. We have computed 78 age trims for each of the three families. These 78 age trims were chosen to cover all possible age trims in which at least five contiguous age groups are used. We picked five as the minimum number of age groups required to give stable estimates for each of the methods. We identify each age trim using the convention *family a–k*, where *family* is either GGB, SEG, or GGBSEG, *a* is the start of the age interval, and *k* is the start of the last 5-y age group included. In this article, we define “method” to mean the specific combination of family and age trim, so effectively we are evaluating three families ×78 age trims = 234 different DDM methods. Application of all methods has been in Stata [22].

### Creating or Identifying Validation Environments

Choice of the optimal DDMs including age trimming can only be undertaken in settings where the analyst has a reasonable knowledge of the true correction factor that needs to be applied to the observed death rate. The real challenge in this research area is creating or identifying existing validation environments. We use three different environments, each with their own advantages: (1) microsimulation model of a population of 10 million followed for a period of 150 y exposed to different levels of age-specific mortality, fertility, and migration. The advantage of the microsimulation environments is that the analyst controls all aspects of population dynamics and measurement error; thus, truth is known with certainty. (2) US counties, 1990–2000, provide a large set of populations with a large range in size, immigration, and emigration rates where it is reasonable to assume that the relative completeness (RC) of vital registration relative to the 1990 and 2000 censuses is close to 100%. (3) High-income OECD economies as designated by the World Bank (July 2009 revision) [23] with populations greater than 5 million from 1950–2000, excluding the Republic of Korea, because registration completeness in past years was not likely complete, and Germany, because no census has been conducted since reunification. This group represents a much narrower range of migration rates and larger population sizes in countries with mature death registration systems.

### Population and Measurement Microsimulation

**Figure 2:**
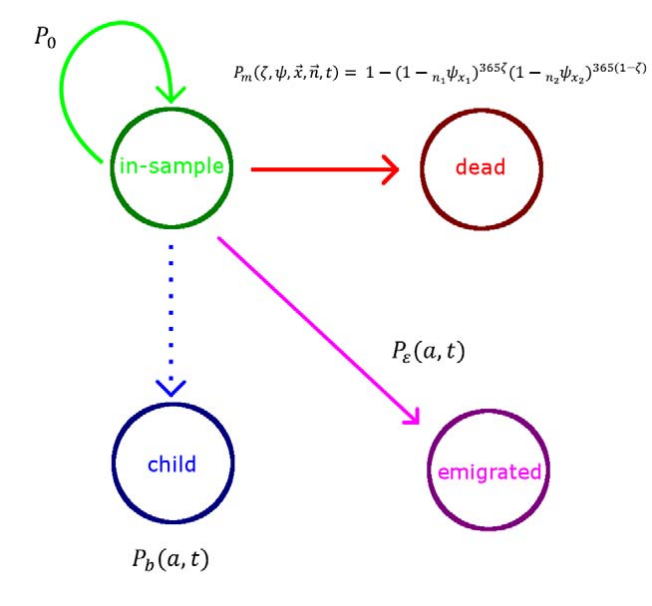
Simulated population model. This schematic describes the evolution of the simulated population, where *P*_0_ is the probability of remaining in the sample, *P*_m_ is the probability of dying in the year given an age-specific probability ψ of dying in a single day (ζ is the fraction of time spent in the year in age group *x*_1_), *P*_ε_(*a*,*t*) is the probability of migrating at age *a* and time *t*, and *P*_b_(*a*,*t*) is the probability of giving birth at age *a* and time *t* and only applies to the reproductive age groups.

**Figure 3:**
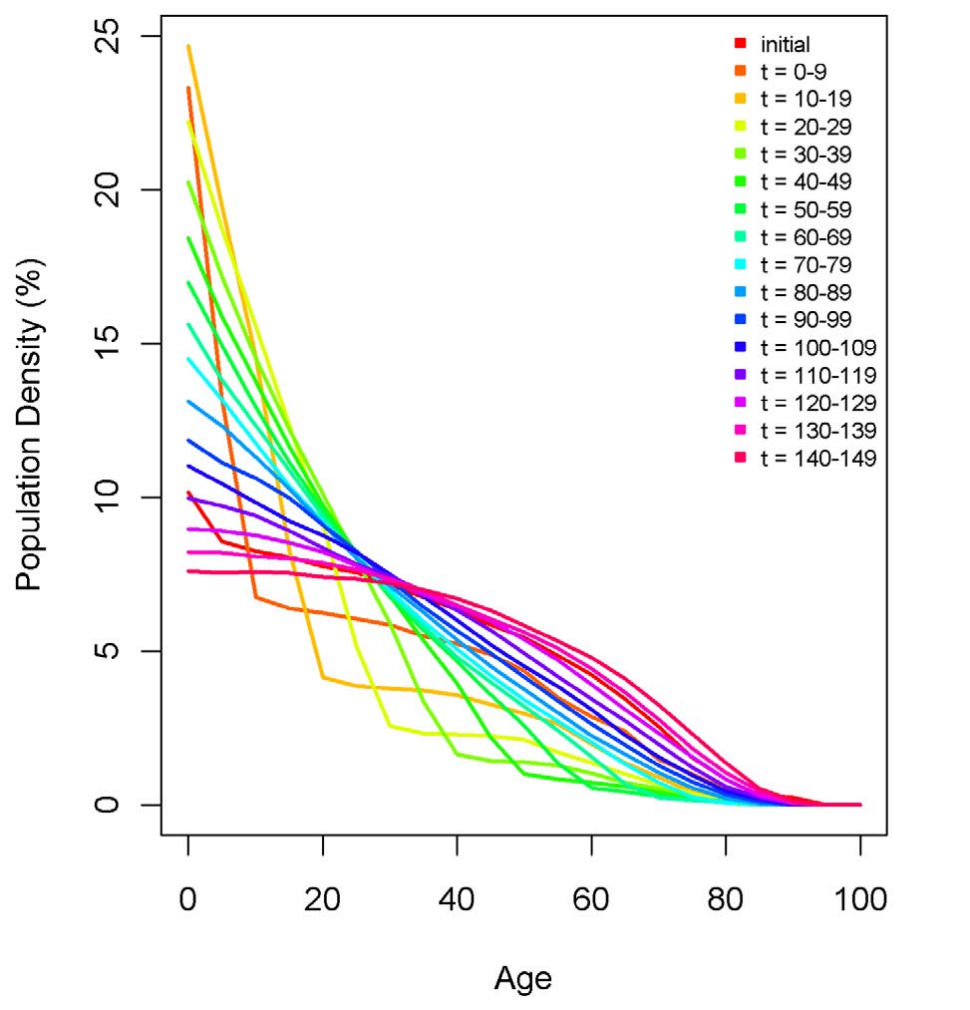
An example of the effect of fertility and mortality evolution over time on the simulated population age-structure with no migration. The initial age structure of Sweden's 1751 population is shown, as are 10-y incremental changes after applying the mortality and fertility rates of the simulated population.

**Figure 4:**
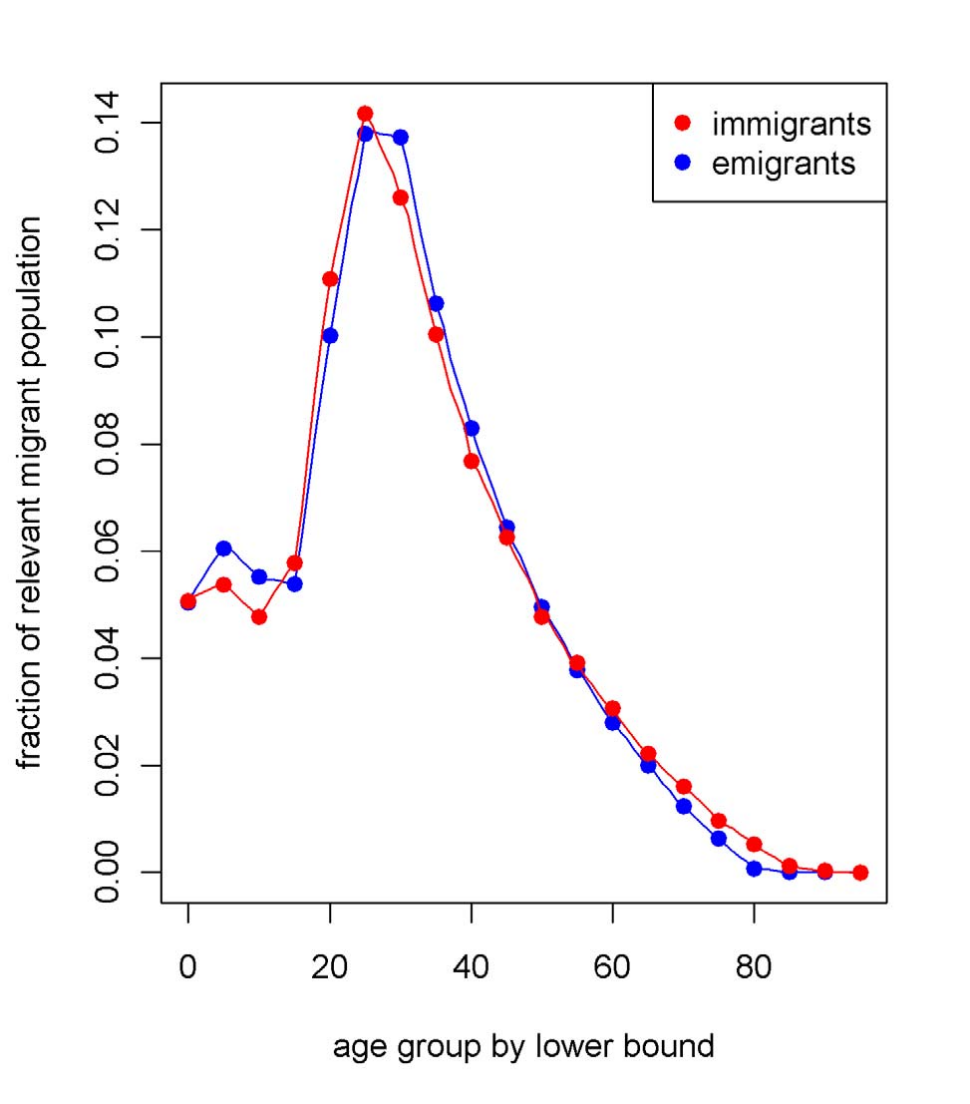
The age pattern of in- and out-migration used to model migration in the simulated populations. This age pattern is based on the average of a geographically diverse selection of countries with complete migration data as reported in the 1989 Demographic Yearbook.

Using microsimulation to study the performance of DDMs requires two interconnected models: a population microsimulation model and a measurement microsimulation model. Figure 2 provides a schematic of the population model where individuals are exposed over time to age-specific risks of death, fertility, and migration. Mortality and fertility rates were modeled on the basis of trends in mortality and fertility in the US during the 20th century. These rates were applied to an initial population age distribution from the year 1751 in Sweden. The effect of fertility and mortality evolution over time on the population age structure is illustrated in Figure 3. After an initial period of approximately 75 y, the age distribution evens out and becomes smooth again. Using this population model, we created 11 different population scenarios on the basis of different levels of mortality and fertility. For each mortality-fertility scenario, we added: three scenarios of net immigration with rates of 5, 10, and 25 per thousand; three scenarios of net emigration with rates of 5, 10, and 25 per thousand; and one scenario with no migration. The age-*pattern* of migration, however (illustrated in Figure 4), is constant in each case of net migration and based on the average of a geographically diverse selection of countries with complete migration data as reported in the 1989 Demographic Yearbook [24]. In all, we generated 77 mortality-fertility-migration population scenarios with data on roughly 10–15 million individuals in each. Various demographic characteristics of each population scenario are shown in Table 1.

**Table 1:** Mortality and fertility levels and trends in the simulations.

Scenario	Crude Birth Rate per 1,000	Life Expectancy at Birth	45q15	Population at Census 1	Population at Census 2	Intercensal Deaths
**1**	15.49	74.82	0.104	81,860	87,052	8,146
**2**	17.53	72.73	0.135	66,723	72,783	6,257
**3**	15.92	75.30	0.096	82,789	88,014	8,157
**4**	16.16	74.89	0.101	80,849	86,251	7,917
**5**	18.19	72.94	0.128	67,433	73,442	6,366
**6**	15.50	74.41	0.107	78,116	83,398	7,695
**7**	18.44	72.26	0.142	63,299	69,228	6,029
**8**	17.76	72.71	0.133	66,473	72,376	6,305
**9**	18.74	71.54	0.154	59,805	65,817	5,668
**10**	17.94	72.42	0.141	65,730	71,903	6,144
**11**	17.87	71.81	0.150	63,059	69,183	5,958

For each population scenario, we applied a measurement microsimulation model where census 1 is taken at time *t*, registration of deaths occurs from time *t* to *t*+10, and census 2 is taken at time *t*+10. Individuals have probabilities of being included in the two censuses of *c1* and *c2* and, if they die, of being registered of *v1*. Further, each individual's age in each measurement is recorded subject to two types of age misreporting: stochastic and systematic. Stochastic age misreporting is captured as a random draw for each individual for each measurement from a normal distribution with mean zero and variance 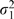. Values selected for the stochastic age-misreporting parameter 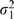 were established by empirical findings from a test-retest comparison of age reporting in 70 World Health Surveys. Systematic age misreporting is captured by the function: 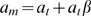 where 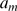 is the misreported age, 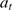 is the true age, and 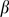 is drawn from a normal distribution with a mean 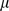 and variance 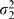. We vary the choice of *c1*, *c2*, *v1*, and the nine parameters defining the age-misreporting distributions 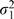 *(c1)*, 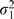 *(c2)*, 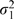 *(vr)*, 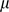 *(c1)*, 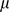 *(c2)*, 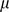 *(vr)*, 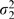 *(c1)*, 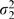 *(c2)*, and 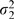 *(vr)*, randomly generating 2,000 different measurement scenarios for each of the 77 population scenarios. Table 2 summarizes the ranges for the nine parameters governing the measurement process that we have used in the simulations. Because we believe that age misreporting is likely to be culturally determined, we have built strong correlations into the selection of age-misreporting variables between any given measurement microsimulations (i.e., age misreporting in one measurement event such as census 1 is similar to age misreporting in another measurement event in the same population). The choice of the ranges sampled in Table 2 is based on our review of the literature [25],[26].

**Table 2:** Simulation measurement model parameter distributions.

Parameter	Mean	Minimum	Maximum
**Completeness of Census 1**	0.95	0.90	1.00
**Completeness of Census 2**	0.95	0.90	1.00
**Completeness of VR**	0.65	0.30	1.00
**β in Census 1**	0.00	−0.07	0.07
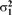 **in Census 1**	1.99	0.00	2.89
**β in Census 2**	0.00	−0.07	0.08
**σ_1_^2^ in Census 2**	1.99	0.00	2.86
**β in VR**	0.00	−0.06	0.07
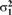 **in VR**	1.99	0.00	2.76

Stochastic age misreporting is captured as a random draw for each individual for each measurement from a normal distribution with mean zero and variance 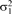. Systematic age misreporting is captured by the function 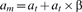 where *a*_m_ is the misreported age, *a*_t_ is the true age, and β is drawn from a normal distribution.

VR, vital registration.

In addition to stochastic and systematic age misreporting, a further aspect to age misreporting is the phenomenon known as “age heaping,” in which respondents tend to favor ages ending in 0 or 5. Because DDMs are applied to 5-y age groupings, we expected the effect of age heaping would be minimal, but we conducted a sensitivity analysis to test this hypothesis. We simulated various degrees of age heaping, in which people rounded their age to the nearest number ending in 5 or 0 with stochastic probabilities of 5%, 10%, or 20%. These values correspond to Whipple's indices of roughly 120, 140, and 180, respectively, and reflect degrees of data quality ranging from “approximate” to “very rough,” according to the United Nations [27].

In total, we have generated 154,000 sets of two censuses and death registration data over 10 y where we know the true death rate and the observed death rate and thus the correction factor that DDMs should generate.

### US Counties 1990–2000

Our second validation environment is US counties 1990 to 2000 where census age counts and death registration are available. We use the 2,072 counties or merged county units developed to assure a minimum population size in each aggregate [28]–[30]. Table 3 summarizes the range of population sizes, mortality, fertility, and migration observed across US counties. We assume that, due to rigorous enforcement of census and registration laws, relative completeness of death registration is effectively 100% in all counties. It is true that census coverage at the county level can vary, but it is estimated to be greater than 98% for close to 90% of all counties [31]. To remove the effect of small numbers on these methods, we present results for the 534 counties with a population greater than 100,000. Internal immigration and emigration data are based on data from the US Internal Revenue Service (IRS) 2007 [32], which tabulates the number of exemptions (an estimate of the number of individuals) that move from each county to every other county by matching the Taxpayer Identification Number and comparing zip codes of filing addresses from one year to the next. The IRS dataset does not include international migrants. The number of international migrants is less than one-quarter of total internal migrants in the United States [32]–[34].

**Table 3:** Summary of demographic characteristics of US counties with population greater than 100,000 in 2000.

Demographic Characteristic	Years	Mean	Minimum	Maximum
**Population size**	2000	401,245	100,224	9,519,338
**Average annual growth rate**	1990–2000	0.015	−0.013	0.103
**Emigration (total outmigrants)**	1995–2000	22,711	547	1,190,823
**Immigration (total inmigrants)**	1995–2000	22,711	226	770,306
**Net migration (net migrants)**	1995–2000	0	−420,571	193,489
**Life expectancy at birth**	1990–2000	78	70	82
**45q15**	1990–2000	0.11	0.06	0.25
**Total fertility rate**	2000	2.01	0	3.62

### High-Income, Large Countries 1950–2000

Our third validation environment, following the work of Thomas and Hill [20], is high-income countries with populations greater than 5 million from 1950–2000. We have identified 149 periods across 20 countries where census data are available at the beginning and end of the period and death registration data are available for part or all of the intermediate years. Periods were defined by pairing a census with each of the two subsequent censuses in time; this yielded two intercensal periods (for example, a census in 1970 would be paired with the 1980 census to create one period and the 1990 census to create a second period; the same would be done with 1980–1990 and 1980–2000 and so on). As required to apply the methods, deaths during the intercensal period were averaged to create average annual deaths within each period. The set of 20 high-income countries consists of: Australia, Austria, Belgium, Canada, Czech Republic, Denmark, Finland, France, Greece, Hungary, Italy, Japan, Netherlands, Portugal, Slovak Republic, Spain, Sweden, Switzerland, the United Kingdom, and the United States. The data sources include the United Nations Demographic Yearbook [35] and the WHO mortality database [36]. We have applied the best performing method for all variants of each family of DDMs to all 149 combinations of censuses and death registration within the matching intercensal period, yielding 149 estimates of relative completeness for each DDM method for these countries. For each of them, we assume, as in the US counties, that relative completeness of death registration is very close to 100%, because social and legal structures in place for several decades mean that it is extremely difficult to dispose of a corpse without legal registration of death.

### Evaluating the Performance of Different DDMs

Each method yields a correction factor that can be multiplied by the observed death rate to get a corrected death rate. Text S1 provides a formula for each of these correction factors by DDM family. For convenience and interpretation, we define “relative completeness of death registration” to be the inverse of the correction factor from each family. It is important to note that the assumptions in each family that are incorporated into the correction factor are different. Nevertheless, we ultimately want DDMs that yield the correction factor closest to the true value needed to correct the observed death rate for a population to equal the true death rate. For ease of communication, we prefer to evaluate DDMs using the inverse of the correction factor, or relative completeness of death registration (RC). For each validation environment, we compare estimated RC to the true or assumed RC. The difference between RC(estimated) and RC(true) is the error in the estimated relative completeness. We use median relative error as a metric of performance of a given method applied in a given validation environment (we use median relative error instead of average relative error because the median is less sensitive to outliers). More formally, we take the median value of:
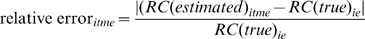
(1)across all *i* for each combination of *tme*. Where *tm* represents the age trim *t* used from family *m* (GGB, SEG, or GGBSEG), *i* indexes each simulated population, county, or country from validation environment *e*. We choose optimal DDMs for each family of methods by minimizing the median relative error in the three validation datasets.

### Diagnostics for DDMs

In practice, demographers often use qualitative insights from the application of DDMs to judge how well they are performing. For example, for GGB, when observed death rates from death registration are plotted against implied death rates from the comparison of censuses, the closer they fall to a straight line, the better the results are considered. For SEG, the relative completeness across age groups should be close to a flat line since the method assumes constant completeness by age. We capture these important insights by formalizing these diagnostics. For GGB, we compute the *R* ^2^ of the regression of implied death rates on observed death rates. If the assumptions of the method hold, then mathematically, these two quantities should fall on a line and *R* ^2^ equal to 1.0. The *R* ^2^ value can be interpreted as a measure of the degree to which the assumptions of the method are upheld or fail. For SEG and GGBSEG, we compute the slope of a regression line of the age-specific relative completeness estimates on age. The closer this slope is to zero the better. For optimal trims of each family of methods, we have explored the relationship between the diagnostics and performance.

### Uncertainty Intervals

We approximated uncertainty intervals by determining, separately in the three environments, the standard error that produced an uncertainty interval that captured the truth 95% of the time. We assumed normality in order to construct the uncertainty intervals.

### Application to Selected Developing Countries

As with the high-income countries, in applying the methods to developing countries, we created periods by pairing each census with the two subsequent censuses. We then applied the optimal age trims for each of the three families of methods to the resulting census pairs and the intercensal average annual deaths from death registration or census/survey data on household deaths found in the UN Demographic Yearbooks [35], IPUMS [37], and WHO [36] mortality databases for 1950–2000. In some cases, the age groups available in the data did not allow for the use of the optimal age trim. For example, if the open interval for deaths was 70+y, we could not apply any age trims above the 65–69-y age group. In these cases, we applied the best performing age trims possible given the age groups present in the dataset. Further, when the death data available were from censuses or surveys that asked about household deaths in the last 12 mo, we computed average annual deaths by calculating the death rates at the time of the second census (or survey), and applying them to the average person-years lived assuming geometric population growth in the intercensal period. In total, this process yielded roughly 1,000 estimates from each optimal DDM. For illustrative purposes, we present our results in detail for six developing countries and contrast them with results from two high-income countries.

## Results

The performance of all possible age trims for the three families of DDMs in the three validation datasets is summarized in Table 4 (which lists the top five and worst five age trims for each method). The full results for every age trim can be found in Table S1. The results in all validation datasets demonstrate high variation in performance across different age trims. This variation ranges from 2.0% to 46% in terms of median relative error. Clearly, the key determinants of the performance of each family of DDMs vary profoundly according to which age groups are included in the estimation process. There is much greater variation across age trims than there is across families of DDMs. As Table 4 shows, we have computed the median relative error for each age trim in each validation environment and ranked the trims within each environment. The minimum average rank across the three environments yields the best performing method. For SEG, the optimal age trim is SEG 55–80; this was the second best in the simulated populations and US counties and best in high-income countries. For GGB, the results across validation datasets appear to be more mixed, but GGB 40–70 performed best on average. Finally, GGBSEG 50–70 performed best on average across the three validation environments.

**Table 4:** Median relative error and rank for the top five and worst five of 78 possible age trims of GGB, SEG, and GGBSEG in the simulations, US counties, and high-income countries sorted by average rank.

Age Trim	Simulations		US Counties		High-Income Countries		Average	
	MRE	Rank	MRE	Rank	MRE	Rank	MRE	Rank
**GGB**								
**40–70**	0.0644	4	0.1086	24	0.02155	7	0.065	11.7
**35–70**	0.0662	5	0.1068	18	0.02255	13	0.065	12.0
**25–75**	0.0696	13	0.1006	13	0.02245	12	0.064	12.7
**30–75**	0.0678	10	0.1007	14	0.02318	14	0.064	12.7
**35–75**	0.0666	7	0.0989	11	0.02467	21	0.063	13.0
**15–35**	0.1274	66	0.4167	74	0.16579	78	0.237	72.7
**20–40**	0.1533	76	0.3773	72	0.13529	73	0.222	73.7
**5–35**	0.144	73	0.41	73	0.14786	77	0.234	74.3
**5–25**	0.2567	78	0.4365	76	0.11805	69	0.270	74.3
**5–30**	0.1875	77	0.4615	77	0.12809	72	0.259	75.3
**SEG**								
**55–80**	0.0869	2	0.1057	2	0.01985	1	0.071	1.7
**60–80**	0.0809	1	0.0984	1	0.02128	4	0.067	2.0
**55–75**	0.0917	3	0.1105	3	0.02035	2	0.074	2.7
**50–80**	0.0938	4	0.1132	4	0.02131	5	0.076	4.3
**45–80**	0.1018	6	0.1224	6	0.02117	3	0.082	5.0
**5–40**	0.2993	74	0.2863	74	0.06559	74	0.217	74.0
**5–35**	0.3141	75	0.2926	75	0.0694	75	0.225	75.0
**10–30**	0.3147	76	0.2975	76	0.07101	76	0.228	76.0
**5–30**	0.329	77	0.3077	77	0.07851	77	0.238	77.0
**5–25**	0.3438	78	0.3263	78	0.0828	78	0.251	78.0
**GGBSEG**								
**50–70**	0.0911	6	0.0917	5	0.03645	8	0.073	6.3
**45–80**	0.0926	7	0.0933	8	0.03588	5	0.074	6.7
**45–70**	0.0967	9	0.0911	4	0.03754	9	0.075	7.3
**50–75**	0.0888	5	0.0947	13	0.03562	4	0.073	7.3
**45–75**	0.0938	8	0.0934	9	0.03645	7	0.075	8.0
**5–40**	0.249	74	0.1866	74	0.09904	74	0.178	74.0
**5–35**	0.2647	76	0.2034	75	0.10833	75	0.192	75.3
**10–30**	0.2619	75	0.2101	76	0.11028	76	0.194	75.7
**5–30**	0.2806	77	0.2154	77	0.11491	77	0.204	77.0
**5–25**	0.296	78	0.2279	78	0.12469	78	0.216	78.0

Because the simulated populations were generated using historical mortality rates from the US, they have slightly older populations on average than low-middle-income countries with vital registration where the DDM methods would be useful. This discrepancy raises the issue as to whether or not the selection of older age trims as the best performing methods might be an artifact of the age structure of the populations. To test this, we used the US county validation environment to examine the optimal age trim across various levels (<10%, 10%–12%, 12%–18%, 18%–20%, and >20%) of the proportion of population above age 60 y (we performed the analysis using the US counties because there was more variation in proportion over age 60 across county populations than in the simulations). The optimal age trims were generally consistent across five categories of proportion over age 60 for the SEG method, and varied more for the GGB and GGBSEG methods. Despite the different optimal age trims across the different categories of population structure for GGB and GGBSEG, there is no consistent evidence to suggest that in more elderly populations, the older age trims are favored.

Given the closest test to national application is the high-income countries, all three optimal versions of the three DDM families perform relatively well with similar median relative error in this setting. SEG 55–80 does slightly better than the optimal age trims in the other families. Of note, the previously reported sensitivity of SEG to migration in high-income countries [20] appears to be largely attenuated in SEG 55–80. We focus on the three optimal methods, SEG 55–80, GGB 40–70, and GGBSEG 50–70, for the rest of the analysis.

The simulation dataset provides an opportunity to investigate how error and estimated relative completeness are associated with factors such as the levels and trends in mortality, fertility, migration, and age misreporting.

We first present the results of the sensitivity analysis to determine if the phenomenon of age heaping affects performance above and beyond the effects of stochastic and systematic age misreporting that we modeled. Table 5 suggests that consistent with our hypothesis, there is no appreciable effect. The optimal age trims remain so for each family, and the median relative error remains essentially the same, regardless of the degree of age heaping. For further analysis of the effects of age misreporting, we focus solely on the effects of stochastic and systematic age-misreporting patterns.

**Table 5:** Age trims with the lowest median relative error in the simulations with and without age heaping.

Order	Without Age Heaping		Age Heaping, 5%		Age Heaping, 10%		Age Heaping, 20%	
	Age Trim	MRE	Age Trim	MRE	Age Trim	MRE	Age Trim	MRE
**GGB**								
**1**	45–70	0.0636	45–70	0.063669	45–70	0.063545	45–70	0.063616
**2**	50–70	0.0639	50–70	0.063898	50–70	0.063869	50–70	0.063791
**3**	45–65	0.0643	40–70	0.064349	40–70	0.06443	40–70	0.064564
**4**	40–70	0.0644	45–65	0.064403	45–65	0.064606	45–65	0.064766
**5**	35–70	0.0662	40–75	0.066096	40–75	0.065963	40–75	0.065847
**SEG**								
**1**	60–80	0.0809	60–80	0.080823	60–80	0.080716	60–80	0.080609
**2**	55–80	0.0869	55–80	0.086894	55–80	0.086842	55–80	0.08681
**3**	55–75	0.0917	55–75	0.09171	55–75	0.091671	55–75	0.09179
**4**	50–80	0.0938	50–80	0.093813	50–80	0.093839	50–80	0.093951
**5**	50–75	0.0991	50–75	0.09913	50–75	0.099233	50–75	0.099424
**GGBSEG**								
**1**	60–80	0.0820	60–80	0.081414	60–80	0.080588	60–80	0.07941
**2**	55–80	0.0846	55–80	0.083923	55–80	0.083185	55–80	0.081934
**3**	55–75	0.0847	55–75	0.083988	55–75	0.083194	55–75	0.082023
**4**	50–80	0.0881	50–80	0.087424	50–80	0.086628	50–80	0.085277
**5**	50–75	0.0888	50–75	0.088041	50–75	0.087206	50–75	0.085958

Table 6 shows regression results of error in relative completeness for each of the three optimal methods regressed on the six age-misreporting variables (stochastic and systematic age-misreporting variables for each of the two censuses and the vital registration system) and migration rate with and without fixed effects for the 77 population scenarios. Overall, the regression results show large effects in all three optimal trims for age misreporting. Stochastic age misreporting has an important effect, but the effect of systematic age misreporting is much larger, judging by the *t*-statistics. Of particular importance are differences in the systematic age-misreporting variables across the two censuses and vital registration (separate regressions not shown). When patterns of age misreporting differ more across the censuses and vital registration (VR), the error increases. Adding population fixed effects increases the *R* ^2^ of the regression, indicating that the parameters defining the population (mortality, fertility, and migration) along with age misreporting together explain a total of 78%, 94%, and 90% of the variation in the error of the SEG 55–80, GGB 40–70, and GGBSEG 50–70 methods, respectively. Notably, the coefficients do not change appreciably when fixed effects are added, indicating that the relationships of age misreporting to relative error can be generalized across different population settings. With fixed effects, migration is not significant as expected because the 77 fixed effects capture the unique effects of the combination of mortality, fertility, and migration. The *t*-statistic on migration in the model without fixed effects is relatively small in comparison to those driven by the error generated by the age-misreporting parameters. In part, this result may be due to the selection of the optimal age trims, which tend to minimize the impact of migration already.

**Table 6:** Coefficients from regression of error on age misreporting and migration in the simulations.

Variables	GGB Estimated	Lower 95% CI	Upper 95% CI	*t*	SEG Estimated	Lower 95% CI	Upper 95% CI	*t*	GGBSEG Estimated	Lower 95% CI	Upper 95% CI	*t*
**(a) Without Fixed Effects**												
**β in Census 1**	−13.00	−13.03	−12.98	−1185.6	−9.81	−9.86	−9.76	−399.9	−15.22	−15.25	−15.19	−986.3
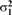 **in Census 1**	−0.02	−0.03	−0.02	−27.4	0.00	0.00	0.01	1.3	−0.04	−0.04	−0.04	−31.8
**β in Census 2**	5.21	5.19	5.23	464.0	5.78	5.74	5.83	230.3	1.52	1.49	1.55	96.3
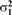 **in Census 2**	0.02	0.02	0.02	24.6	0.02	0.02	0.02	10.0	0.02	0.02	0.03	19.3
**β in VR**	8.68	8.65	8.70	769.6	4.84	4.79	4.89	191.8	14.10	14.07	14.13	889.0
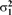 **in VR**	0.00	0.00	0.01	4.7	−0.02	−0.03	−0.02	−12.4	0.02	0.01	0.02	12.2
**Migrants per 100,000**	−0.14	−0.14	−0.14	−237.8	0.37	0.37	0.37	284.1	−0.03	−0.04	−0.03	−40.9
**Constant**	0.03	0.03	0.03	50.2	0.02	0.02	0.02	14.8	0.05	0.04	0.05	58.0
***R*** **^2^**	0.91	—	—	—	0.63	—	—	—	0.89	—	—	—
**RMSE**	0.03	—	—	—	0.07	—	—	—	0.05	—	—	—
**(b) Including Fixed Effects for Population Scenario**												
**β in Census 1**	−13.00	−13.02	−12.99	−1484.0	−9.81	−9.85	−9.78	−521.4	−15.22	−15.25	−15.19	−1069.5
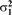 **in Census 1**	−0.02	−0.03	−0.02	−34.3	0.00	0.00	0.01	1.7	−0.04	−0.04	−0.04	−34.4
**β in Census 2**	5.21	5.19	5.23	580.8	5.78	5.75	5.82	300.3	1.52	1.49	1.55	104.4
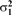 **in Census 2**	0.02	0.02	0.02	30.7	0.02	0.02	0.02	13.1	0.02	0.02	0.03	20.9
**β in VR**	8.68	8.66	8.69	963.3	4.84	4.80	4.87	250.1	14.10	14.07	14.13	964.0
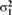 **in VR**	0.00	0.00	0.01	5.8	−0.02	−0.03	−0.02	−16.1	0.02	0.01	0.02	13.2
**Migrants per 100,000**	−0.22	−0.23	−0.22	−65.7	0.57	0.56	0.59	79.1	−0.07	−0.08	−0.06	−12.8
**Constant**	0.03	0.03	0.03	36.4	0.02	0.02	0.02	12.3	0.03	0.03	0.04	28.6
***R*** **^2^**	0.94	—	—	—	0.78	—	—	—	0.90	—	—	—
**RMSE**	0.03	—	—	—	0.06	—	—	—	0.04	—	—	—

This table shows the relationship between levels of age-misreporting and migration and error in relative completeness (RC) in the simulation environment, both in the absence (a) and presence (b) of fixed effects, indicating the combination of mortality, fertility, and migration rates that define a population scenario. Error is calculated by dividing the difference between true RC and estimated RC by true RC using the optimal variant in the simulated environment for each of the three families. Stochastic age-misreporting is captured as a random draw for each individual from a normal distribution with mean zero and variance 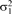. Systematic age-misreporting is captured by the function 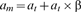 where *a*_m_ is the misreported age, *a*_t_ is the true age, and β is drawn from a normal distribution.

CI, confidence interval; RMSE, root mean squared error; VR, vital registration.

We also analyzed the performance of the age trims in the simulations and US counties according to level of migration. Table 7 shows the optimal age trim at each level of migration for the simulation, and Table 8 at each quintile of migration for the counties. The best performing age trims, as measured by median relative error, are the same across varying levels of migration for SEG and GGBSEG, and they are only slightly different for GGB. For SEG and GGBSEG, the age trim 60–80 has the smallest median relative error in all migration scenarios in the simulations. For GGB, in the absence of net migration, the age trim 40–60 has the smallest median relative error, whereas the age trims 45–65 and 45–70 are the better performers under positive or negative net migration scenarios. In the US county environment, the best age trims for each method are fairly consistent across migration levels. The same age trim (60–80) is best for all quintiles of migration for SEG. The optimal age trim varies a bit more for the first quintile of GGB (25–80 versus 55–80 and 60–80 for the other categories) and across each of the categories for GGBSEG (generally, younger age groups are included in the age trim with less migration present; however, the change in age groups is not dramatic). These results suggest that the performance of the optimal methods is relatively immune to different levels of migration.

**Table 7:** Age trims with the lowest median relative error for different levels of migration in the simulations.

Migrants per 1,000	Best Age Trim (y)
**GGB**	
**−25**	45–70
**−10**	45–70
**−5**	45–65
**0**	40–60
**5**	45–65
**10**	45–65
**−25**	45–70
**SEG**	
**−25**	60–80
**−10**	60–80
**−5**	60–80
**0**	60–80
**5**	60–80
**10**	60–80
**−25**	60–80
**GGBSEG**	
**−25**	60–80
**−10**	60–80
**−5**	60–80
**0**	60–80
**5**	60–80
**10**	60–80
**−25**	60–80

**Table 8:** Age trims with the lowest median relative error for different quintiles of migration in US counties.

Quintile	Migrants per 1,000	Best Age Trim (y)
**GGB**		
**1st**	[−95.7, −23.0)	25–80
**2nd**	[−23.0, −7.35)	55–80
**3rd**	[−7.35, 10.6)	60–80
**4th**	[10.6, 39.0)	60–80
**5th**	[39, 276]	60–80
**SEG**		
**1st**	[−95.7, −23.0)	60–80
**2nd**	[−23.0, −7.35)	60–80
**3rd**	[−7.35, 10.6)	60–80
**4th**	[10.6, 39.0)	60–80
**5th**	[39, 276]	60–80
**GGBSEG**		
**1st**	[−95.7, −23.0)	50–70
**2nd**	[−23.0, −7.35)	40–75
**3rd**	[−7.35, 10.6)	30–55
**4th**	[10.6, 39.0)	40–70
**5th**	[39, 276]	45–65

**Figure 5:**
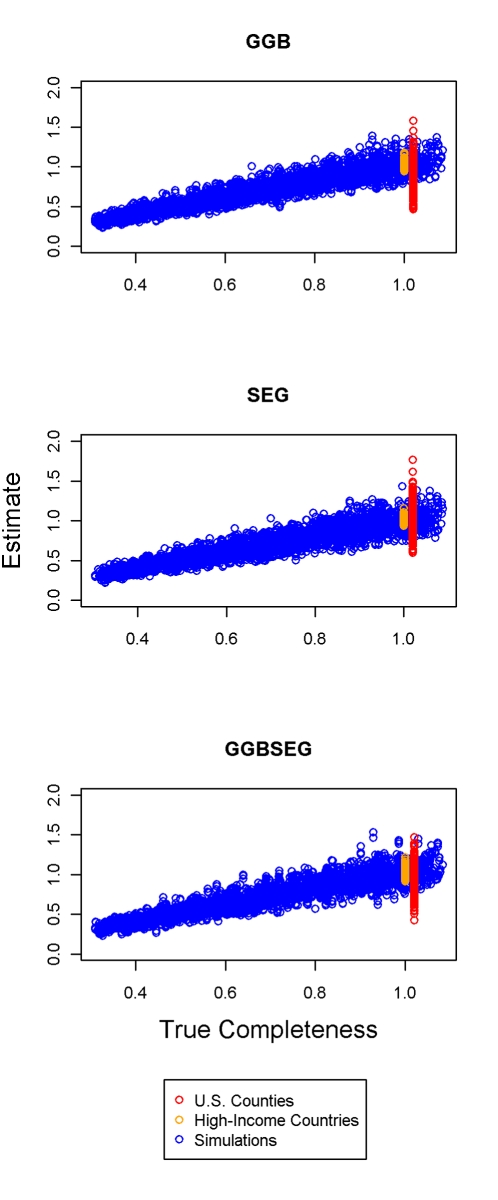
Estimated relative completeness versus true relative completeness in the simulations, US counties with a population greater than 100,000, and large high-income countries for the three methods. The distribution of true completeness for the US counties is artificially offset from 1 in order to better distinguish it graphically from high-income countries.

Uncertainty in the estimated relative completeness is large. Figure 5 shows in the three validation datasets the relationship between the estimated relative completeness and the true relative completeness for the optimal method in each of the three families. The variation in estimated relative completeness increases as true coverage approaches 100%. When the error in relative completeness is expressed in relative terms by dividing by the true level of relative completeness, error remains constant as a function of true completeness (unpublished data). Because Figure 5 shows that uncertainty is a function of the level of completeness, the appropriate way to express uncertainty for these methods is in terms of variation relative to the estimated completeness. In other words, we compare the standard deviations of the distributions of relative error that generate confidence intervals capturing 95% of the true values. The uncertainty interval for the high-income countries is smallest; we found standard errors of 0.05, 0.037, and 0.07 for GGB, SEG, and GGBSEG, respectively. In US counties with a population over 100,000, the standard errors were 0.24, 0.16, and 0.15 for the three methods. The standard errors in the simulations were more similar to those in the US counties: 0.10 for GGB, 0.13 for SEG, and 0.13 for GGBSEG. The range of population dynamics and data generation errors is quite varied across each of these environments. We suggest taking the conservative estimate of ±20%–26% from the simulation results because this range falls between the uncertainties estimated from the counties, which may be overestimating uncertainty because of smaller numbers and higher levels of migration, and the high-income countries, which are likely underestimating the uncertainty we would expect in an application to data from low- or middle-income countries.

**Figure 6:**
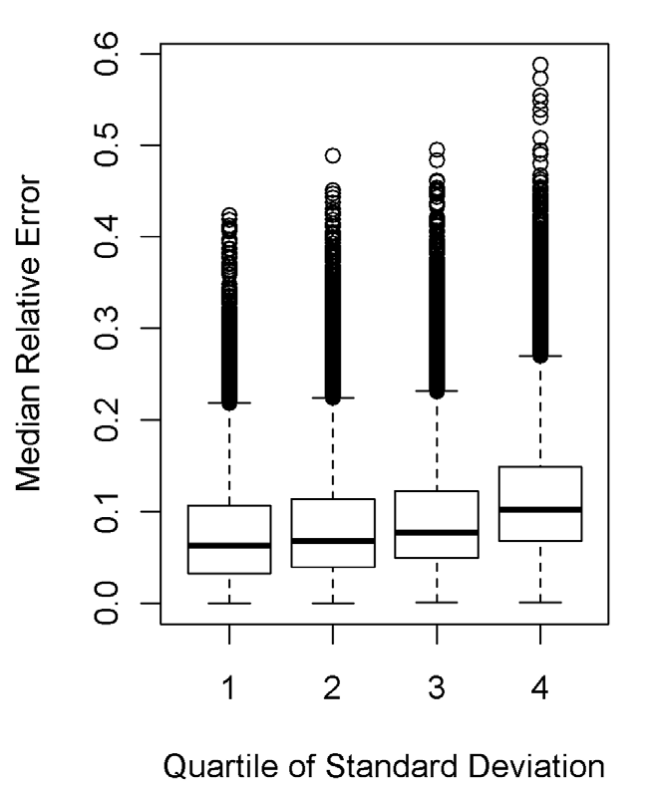
Relationship between median relative error and quartile of variance across (inconsistency between) the three families of DDM methods.

We explored the relationship between the variation or inconsistency across the optimal age trims from each family and the resultant error. More specifically, we examined the error in each of the three methods by quartiles of the variance. While there was an overall relationship (Figure 6), there was much variability indicating that greater consistency was not always indicative of less error.

**Figure 7:**
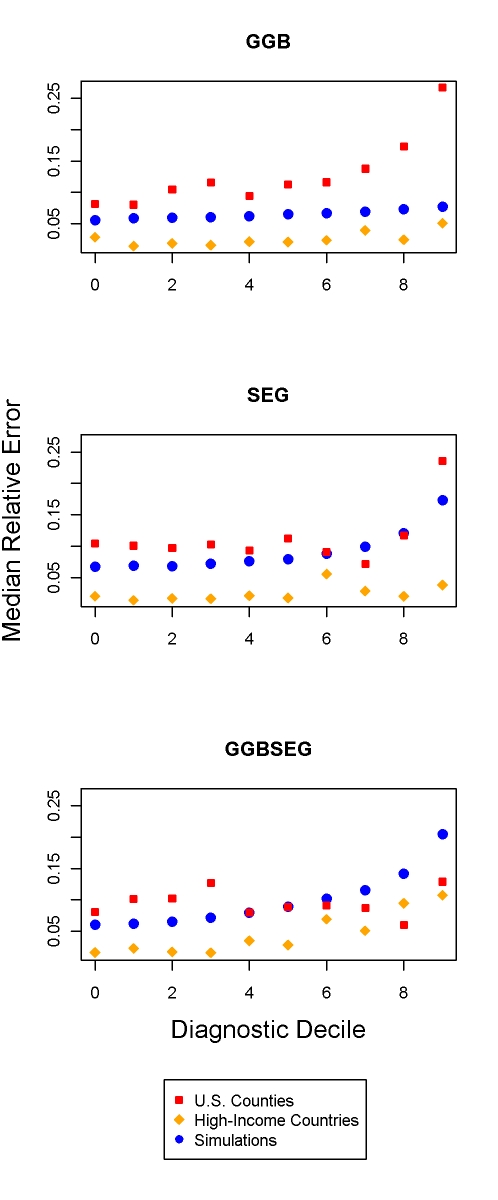
Relationship between median relative error and decile of diagnostics for GGB, SEG, and GGBSEG. For GGB, the diagnostic is the *R* ^2^ of the regression of observed death rates on implied death rates. For SEG and GGBSEG, the diagnostic is the slope of the regression of age-specific completeness estimates on age.

Diagnostics for GGB 40–70, SEG 55–80, and GGBSEG 50–70 may provide an indication of when methods are performing better or worse. In the three validation datasets, we examined the relationship between performance and low to high values of the diagnostics (Figure 7). Performance is somewhat related to the diagnostics, more so in the US counties than in the simulations or high-income countries for GGB and the reverse for GGBSEG. For SEG, only extreme values of the diagnostics are related to error for the US counties, and there appears to be a slight relationship with error in the simulations. No clear relationship is evident in the high-income countries. Given the different relationships across each of the methods, the fact that the relationship between diagnostic decile and error is not always linear, and that the error tends to be responsive only to extreme values of the diagnostics, use of these indicators to assess performance does not appear to be very informative.

**Figure 8:**
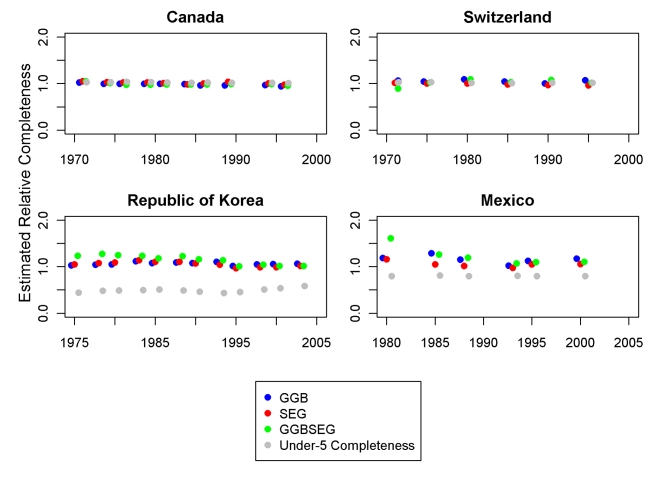
Application of optimal DDMs to Canada, Switzerland, Korea, and Mexico.

**Figure 9:**
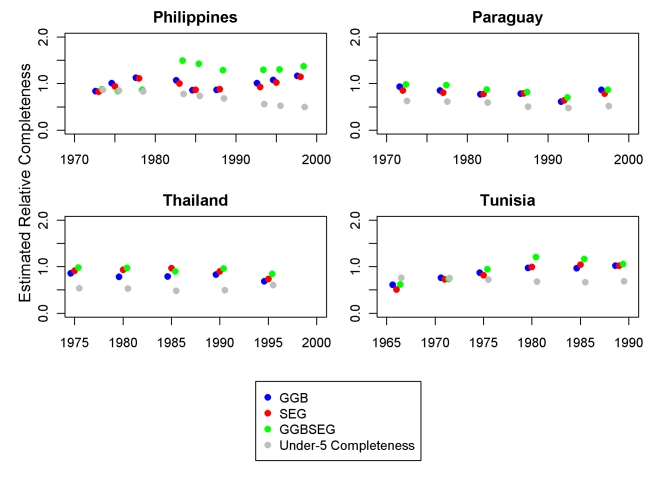
Application of optimal DDMs to the Philippines, Paraguay, Thailand, and Tunisia.

Figures 8 and 9 show eight examples of the application of the three optimal methods to select countries over time. In each, for each pair of censuses and vital registration data, we show the results of the three methods in terms of relative completeness. For comparison, we have also compared registered deaths 0–4 to estimates of 0–4 deaths on the basis of systematic review of all data sources [1]. We include graphs from two high-income countries, Canada and Switzerland, to show that these methods can be consistent and accurate, as we assume death registration is complete in these countries. The graph for Mexico suggests that vital registration, at least for adults, has been relatively complete since 1970, but there is clearly more noise in these estimates than for the high-income countries shown. The Philippines shows similarly noisy estimates, with both decline and improvement suggested. Knowing the uncertainty inherent in these methods, however, it is unclear that these are true trends. In Thailand, registration for adults is estimated to be between 72% and 98% complete during the 1980s and 1990s. Paraguay is another example where registration completeness has been relatively constant over time and where the methods seem to be fairly consistent with one another. Tunisia illustrates an example where death registration has clearly improved over time, from nearly 50% in 1965 to complete by 1980. Finally, the graph for Korea shows complete registration over time for adults, though child completeness is notably less. The variation in the estimates across methods decreases over time. Across all the countries, it is not clear that any one family of DDMs is best or most consistent, although SEG appears to be slightly more stable.

## Discussion

On the basis of a systematic evaluation of the performance of 234 variants of DDM methods on three different validation datasets where we know or have strong beliefs about the true level of completeness of death registration, we have identified improved DDM methods, characterized their uncertainty in different settings, and illustrated their applicability in developing countries.

This thorough analysis of the three families of DDMs whose purpose is to estimate the completeness of death registration demonstrates that the choice of age trims has a profound effect on the performance of these methods. On the basis of the three different validation datasets, we believe SEG 55–80, GGB 40–70, and GGBSEG 50–70 are the best methods that can be currently used to estimate relative completeness of death registration. The combination of the three optimal DDMs will yield much better results than the current practice of application of DDMs without optimal age trimming. Selection of optimal age trims has also substantially reduced the bias associated with migration reported in previous work.

Though there is an overall relationship between greater variation across the estimates from GGB, SEG, and GGBSEG and greater relative error, much variation exists in this relationship. We feel it is best to interpret the results from the three best methods in the context of other information, such as independent estimates of under-5 completeness and DDM results over time, and there is an avenue of future research to determine a systematic way of doing so. However, if an estimate of completeness must be obtained solely from the optimal DDMs at time period, then the median result across these three best age trims can be used. Using the median yields the second lowest median relative error in all three validation environments, whereas the best age trims from the other families alone do not perform as consistently well across validation environments (unpublished data). It also performs well and most consistently in terms of minimizing the standard error required to construct an uncertainty interval that captures the truth 95% of the time.

Published studies and national statistical reports apply these methods and provide results without uncertainty bounds. In the three different validation environments, our results indicate that estimated relative completeness using the optimal age trims has a minimum median relative error of 2.0%, and this can be as high as 10.9%. Further, one cannot depend on diagnostics to tell the analyst when the uncertainty may be smaller. It appears that the underlying stochastic processes in censuses and death registration, including age misreporting, have led to a component of uncertainty that cannot be eliminated. While usage of partial death registration is useful for estimating mortality levels among adults, the application of DDMs, even from the optimal age trims we have suggested here, should be interpreted with considerable caution; the uncertainty around relative completeness of registration is likely to be at least +/−20% of the estimated level, and perhaps considerably more. This level of uncertainty is likely to mean that while DDM correction methods could be useful in estimating levels, they are unlikely to be as useful for estimating mortality change. As an example, Lopez and colleagues [38] estimate that *45q15* (the probability that a 15-y-old would die by age 60 if mortality rates remained constant—a commonly used summary measure of adult mortality) for females in Paraguay declined by 8 per 1,000 over the period of 1990–2001. According to our analysis, a margin of error of 0.21 for the SEG 55–80 estimated relative completeness of 80% for Paraguay in the late 1990s (0.133 standard error for SEG 50–80×1.96×0.8 estimated relative completeness = 0.21) would yield an uncertainty interval around predicted *45q15* between 84 and 143 per 1,000, a spread of 59 points. Detecting the decline in adult female mortality that is estimated to have happened in Paraguay during the period 1990–2001 would not be possible given the uncertainty inherent in the DDMs.

Our working hypotheses in applied work have been that (1) the completeness of adult death registration is usually greater than or equal to the completeness of child death registration, given the greater ease of disposing of infant or child remains without notice of legal authorities compared to those of adults. In addition, we have operated under the assumption that (2) the evolution of social and public institutions leads to stronger civil registration that will improve both adult and child death registration and thus generate a high correlation between adult and child completeness. Application of our optimal DDMs, however, provides indications that assumption (2) may not be entirely accurate. There are a number of developing countries in Latin America and Southeast Asia where adult registration appears to be complete, but child registration varies from complete to less than 50%. There may well be considerable variation across countries in the time lag between achieving complete or near complete adult death registration and the same for children.

Given the residual uncertainty in optimal DDMs, there may be a bigger role for direct measurement of relative completeness through surveys or censuses. Two methods deserve broader consideration. First, some surveys, such as Thailand's 1995–1996 Survey of Population Change [39], have asked households about deaths in the last 12 mo and whether the death was registered, a direct assessment of completeness of death registration. Of course, it is possible that in countries where death registration is legally required, the reported levels of death registration from such a survey may be inflated. Nevertheless, this avenue of measurement could be further refined to include a validation component. Household respondents could be asked if deaths also occurred in hospital, for example. The number of hospital deaths recorded by the health information system could be examined to cross-validate household responses. A second strategy would be to apply capture-recapture or dual-record methods [40],[41] to civil registration deaths and deaths reported by households in a time period prior to a survey or census. Capture-recapture methods require matching of individual deaths, so this effort can be time consuming. Direct measurements of completeness using this approach have been used in the Chinese Disease Surveillance Point System [42] and at Demographic Surveillance Sites in Kenya [43], as well as with recent work in Thailand with the most recent 2006 Survey of Population Change (P. Prasartkul, P. Vapattanawong, personal communication). More experience with both types of approaches may strengthen our capacity to track the completeness of death registration.

The analysis in simulated populations of the profound impact of stochastic and systematic age misreporting has a more general implication. Preston and others [44] have pointed out that even in complete death registration systems, age misreporting can bias the measurement of death rates by age. In a typical developing country with a young age structure, even stochastic age misreporting will lead to overestimation of death rates at younger ages and underestimation of death rates at older ages. The sensitivity of completeness estimates from DDMs to age misreporting compounds this problem. Renewed efforts will be needed to measure the extent of stochastic and systematic age misreporting and provide tools for correcting the bias in observed death rates. This bias is likely present in all available national life tables at present.

Given the increasing availability of other measurements of adult mortality such as corrected sibling survival, corrected death registration data should be interpreted in the context of all other data sources. In the arena of child mortality, it is now standard practice [1] to examine all data sources for a country over time and generate a composite estimate of levels and trends in child mortality. We believe with improved DDMs, there continues to be a role for partial vital registration data in measuring adult mortality levels and trends. But such results should only be interpreted alongside all other data sources on adult mortality and the face validity of the resulting levels, trends, and age-patterns of adult death considered.

## Supporting Information

Table S1 Median relative error and rank for all possible age trims and each DDM family of methods in the simulations, US counties, and high-income countries, sorted by average rank.
(0.25 MB PDF)

Text S1 Summary of DDMs.
(0.07 MB PDF)

## Abbreviations

DDM: death distribution method
GGB: generalized growth balance
GGBSEG: generalized growth balance-synthetic extinct generation
RC: relative completeness
SEG: synthetic extinct generation
