# Supplementary material for: What Can We Conclude from Death Registration? Improved Methods for Evaluating Completeness
Source: PLoS Med. 2010 Apr 13;7(4):e1000262. doi: 10.1371/journal.pmed.1000262 (PMC2854130; doi:10.1371/journal.pmed.1000262)
Supplement: Table S1 — Median relative error and rank for all possible age trims and each DDM family of methods in the simulations, US counties, and high-income countries, sorted by average rank. (0.25 MB PDF) [file pmed.1000262.s001.pdf]

Supporting Information Table S1: Median relative error (MRE) and rank for all possible age-trims and each DDM family of methods in the simulations, US counties, and high-income countries, sorted by average rank.

GGB

	Simulations		US Counties		High-Income Countries		Average	
Age Trim	MRE	Rank	MRE	Rank	MRE	Rank	MRE	Rank
40to70	0.0644	4	0.1086	24	0.02155	7	0.065	11.7
35to70	0.0662	5	0.1068	18	0.02255	13	0.065	12.0
25to75	0.0696	13	0.1006	13	0.02245	12	0.064	12.7
30to75	0.0678	10	0.1007	14	0.02318	14	0.064	12.7
35to75	0.0666	7	0.0989	11	0.02467	21	0.063	13.0
30to70	0.0693	12	0.1072	20	0.02194	10	0.066	14.0
10to80	0.0747	29	0.1	12	0.02049	2	0.065	14.3
20to75	0.0713	16	0.107	19	0.02178	8	0.067	14.3
50to75	0.068	11	0.1076	21	0.02206	11	0.066	14.3
25to80	0.0736	22	0.0954	4	0.02412	17	0.064	14.3
15to80	0.0745	28	0.0966	7	0.02191	9	0.064	14.7
20to80	0.0741	25	0.0959	5	0.02352	15	0.065	15.0
5to80	0.0744	27	0.1046	16	0.02098	4	0.067	15.7
45to75	0.0667	9	0.1083	23	0.02357	16	0.066	16.0
15to75	0.0727	17	0.1121	26	0.0212	5	0.069	16.0
35to80	0.0737	23	0.0965	6	0.02454	19	0.065	16.0
40to75	0.0663	6	0.1029	15	0.02511	28	0.065	16.3
25to70	0.0729	18	0.1185	31	0.02003	1	0.071	16.7
10to75	0.0735	21	0.1136	28	0.0207	3	0.069	17.3
40to65	0.0666	8	0.11	25	0.02463	20	0.067	17.7
5to75	0.0734	19	0.1141	29	0.02139	6	0.070	18.0
35to65	0.0709	15	0.1082	22	0.02446	18	0.068	18.3
45to70	0.0636	1	0.1191	32	0.02497	24	0.069	19.0
30to80	0.0735	20	0.0969	8	0.02531	29	0.065	19.0
55to75	0.0697	14	0.1067	17	0.02509	27	0.067	19.3
40to80	0.0744	26	0.0972	9	0.02589	30	0.066	21.7
45to65	0.0643	3	0.1162	30	0.02672	33	0.069	22.0
50to80	0.0777	33	0.0954	3	0.02706	34	0.067	23.3
45to80	0.0758	30	0.0977	10	0.02669	31	0.067	23.7
50to70	0.0639	2	0.1209	34	0.0273	35	0.071	23.7
55to80	0.0804	37	0.092	2	0.02736	36	0.067	25.0
60to80	0.0842	40	0.0872	1	0.02879	40	0.067	27.0
20to70	0.0762	31	0.1287	36	0.0249	22	0.077	29.7
30to65	0.0765	32	0.1207	33	0.02509	26	0.074	30.3
40to60	0.0741	24	0.1134	27	0.02892	41	0.072	30.7
10to70	0.0797	36	0.1298	37	0.02493	23	0.078	32.0
15to70	0.0785	34	0.1301	38	0.02506	25	0.078	32.3

GGB

Age Trim	Simulations MRE	Simulations Rank	US Counties MRE	US Counties Rank	High-Income Countries MRE	High-Income Countries Rank	Average MRE	Average Rank
5to70	0.0791	35	0.1379	39	0.02672	32	0.081	35.3
35to60	0.081	38	0.1269	35	0.02768	37	0.079	36.7
25to65	0.082	39	0.138	40	0.03058	43	0.084	40.7
30to60	0.0889	44	0.1463	42	0.0282	38	0.088	41.3
20to65	0.0861	41	0.149	44	0.02961	42	0.088	42.3
15to65	0.0884	43	0.1482	43	0.03105	44	0.089	43.3
25to60	0.0955	47	0.1587	47	0.02837	39	0.094	44.3
35to55	0.0946	46	0.1396	41	0.03549	47	0.090	44.7
10to65	0.0892	45	0.1505	45	0.03192	45	0.091	45.0
5to65	0.0882	42	0.1567	46	0.03588	49	0.094	45.7
20to60	0.0996	50	0.1704	48	0.03323	46	0.101	48.0
30to55	0.1044	53	0.1746	50	0.03574	48	0.105	50.3
15to60	0.1012	52	0.1735	49	0.04042	51	0.105	50.7
5to60	0.0993	49	0.1873	52	0.04341	53	0.110	51.3
10to60	0.101	51	0.1804	51	0.04112	52	0.108	51.3
25to55	0.1116	56	0.1899	53	0.03993	50	0.114	53.0
20to55	0.1153	58	0.2094	55	0.04667	55	0.124	56.0
15to55	0.1159	59	0.2087	54	0.05333	56	0.126	56.3
10to55	0.114	57	0.2143	56	0.05561	57	0.128	56.7
5to55	0.1106	55	0.2244	58	0.06272	59	0.133	57.3
30to50	0.1236	63	0.2155	57	0.0452	54	0.128	58.0
25to50	0.1307	68	0.2405	59	0.06131	58	0.144	61.7
5to50	0.1185	60	0.2682	63	0.08005	63	0.156	62.0
10to50	0.1254	65	0.2575	61	0.07692	62	0.153	62.7
15to50	0.1306	67	0.2508	60	0.07574	61	0.152	62.7
20to50	0.1329	70	0.2609	62	0.06602	60	0.153	64.0
5to45	0.1214	61	0.3163	67	0.10529	68	0.181	65.3
10to30	0.0973	48	0.5085	78	0.13932	75	0.248	67.0
10to45	0.1308	69	0.3107	66	0.10274	67	0.181	67.3
15to45	0.1411	72	0.3035	65	0.10234	66	0.182	67.7
25to45	0.1496	75	0.3025	64	0.08778	64	0.180	67.7
10to40	0.1237	64	0.3616	70	0.12037	70	0.202	68.0
10to35	0.11	54	0.4292	75	0.14667	76	0.229	68.3

GGB

	Simulations		US Counties		High-Income Countries		Average	
Age Trim	MRE	Rank	MRE	Rank	MRE	Rank	MRE	Rank
5to40	0.123	62	0.366	71	0.13828	74	0.209	69.0
20to45	0.148	74	0.3188	68	0.10003	65	0.189	69.0
15to40	0.1395	71	0.3502	69	0.12473	71	0.205	70.3
15to35	0.1274	66	0.4167	74	0.16579	78	0.237	72.7
20to40	0.1533	76	0.3773	72	0.13529	73	0.222	73.7
5to35	0.144	73	0.41	73	0.14786	77	0.234	74.3
5to25	0.2567	78	0.4365	76	0.11805	69	0.270	74.3
5to30	0.1875	77	0.4615	77	0.12809	72	0.259	75.3

SEG

	Simulations		US Counties		High-Income Countries		Average	
Age Trim	MRE	Rank	MRE	Rank	MRE	Rank	MRE	Rank
55to80	0.0869	2	0.1057	2	0.01985	1	0.071	1.7
60to80	0.0809	1	0.0984	1	0.02128	4	0.067	2.0
55to75	0.0917	3	0.1105	3	0.02035	2	0.074	2.7
50to80	0.0938	4	0.1132	4	0.02131	5	0.076	4.3
45to80	0.1018	6	0.1224	6	0.02117	3	0.082	5.0
50to75	0.0991	5	0.1199	5	0.02206	6	0.080	5.3
50to70	0.1051	7	0.127	7	0.02335	8	0.085	7.3
45to75	0.1076	8	0.1285	8	0.0233	7	0.086	7.7
40to80	0.1109	9	0.129	9	0.0238	9	0.088	9.0
45to70	0.1141	10	0.1326	10	0.0238	10	0.090	10.0
45to65	0.1213	12	0.1422	13	0.02509	11	0.096	12.0
40to75	0.1172	11	0.1337	11	0.02594	15	0.092	12.3
35to80	0.1213	13	0.1402	12	0.02585	14	0.096	13.0
30to80	0.1329	17	0.1462	16	0.02527	12	0.101	15.0
40to70	0.1244	14	0.1444	14	0.02638	18	0.098	15.3
35to75	0.1282	15	0.1459	15	0.02694	19	0.100	16.3
30to75	0.1407	19	0.1523	18	0.02544	13	0.106	16.7
35to70	0.1361	18	0.1522	17	0.02629	17	0.105	17.3
40to65	0.1321	16	0.1534	19	0.02789	24	0.104	19.7
40to60	0.1411	20	0.1629	23	0.02599	16	0.110	19.7
35to65	0.1448	21	0.1611	22	0.02729	21	0.111	21.3
25to80	0.1453	22	0.1534	20	0.02806	26	0.109	22.7
30to70	0.1492	23	0.1595	21	0.02799	25	0.112	23.0
30to65	0.1583	27	0.1673	26	0.02748	22	0.118	25.0
35to60	0.1544	25	0.1727	27	0.02841	27	0.118	26.3
30to60	0.1683	31	0.1802	31	0.02729	20	0.125	27.3
25to75	0.1539	24	0.1632	24	0.02981	35	0.116	27.7
25to70	0.1627	28	0.1728	28	0.02851	30	0.121	28.7
35to55	0.1647	29	0.1815	32	0.02907	31	0.125	30.7
20to80	0.158	26	0.1649	25	0.03121	42	0.118	31.0
25to65	0.1722	33	0.1833	33	0.02844	28	0.128	31.3
20to75	0.1667	30	0.1753	29	0.03023	37	0.124	32.0
20to70	0.176	34	0.1862	35	0.02844	29	0.130	32.7
15to80	0.1705	32	0.1792	30	0.03074	39	0.127	33.7
25to60	0.1827	38	0.1927	41	0.02778	23	0.134	34.0
30to55	0.1791	35	0.1918	39	0.02914	32	0.133	35.3
15to75	0.1794	36	0.1841	34	0.0305	38	0.131	36.0

SEG

	Simulations		US Counties		High-Income Countries		Average	
Age Trim	MRE	Rank	MRE	Rank	MRE	Rank	MRE	Rank
20to65	0.186	39	0.1915	38	0.02925	33	0.136	36.7
15to70	0.1892	40	0.191	37	0.03118	41	0.137	39.3
10to80	0.1825	37	0.1877	36	0.03386	47	0.135	40.0
25to55	0.1942	43	0.2013	44	0.03023	36	0.142	41.0
30to50	0.1914	41	0.2003	43	0.03099	40	0.141	41.3
20to60	0.1973	45	0.203	46	0.02931	34	0.143	41.7
10to75	0.1922	42	0.1923	40	0.0335	46	0.139	42.7
15to65	0.2	46	0.2023	45	0.03252	45	0.145	45.3
5to80	0.1968	44	0.1991	42	0.0368	53	0.144	46.3
25to50	0.2073	49	0.2139	50	0.03182	43	0.151	47.3
20to55	0.2096	50	0.2171	52	0.03227	44	0.153	48.7
10to70	0.2026	47	0.2048	47	0.03588	52	0.148	48.7
15to60	0.2118	51	0.2115	48	0.03412	49	0.152	49.3
5to75	0.2069	48	0.212	49	0.03793	57	0.152	51.3
10to65	0.2137	52	0.215	51	0.03691	54	0.155	52.3
20to50	0.2232	55	0.2284	57	0.03397	48	0.162	53.3
25to45	0.2219	54	0.2272	56	0.03444	50	0.161	53.3
15to55	0.2244	56	0.2222	54	0.03557	51	0.161	53.7
5to70	0.2173	53	0.2198	53	0.04013	60	0.159	55.3
10to60	0.2256	57	0.2265	55	0.03771	55	0.163	55.7
15to50	0.2378	59	0.2354	60	0.03773	56	0.170	58.3
5to65	0.2288	58	0.2297	58	0.04026	61	0.166	59.0
10to55	0.2382	61	0.2344	59	0.03883	59	0.170	59.7
20to45	0.2379	60	0.2388	62	0.0384	58	0.172	60.0
5to60	0.2415	62	0.2383	61	0.04338	65	0.174	62.7
10to50	0.2522	63	0.2464	63	0.04235	63	0.180	63.0
15to45	0.2531	64	0.2488	65	0.04282	64	0.182	64.3
20to40	0.2542	65	0.2547	66	0.04191	62	0.184	64.3
5to55	0.255	66	0.2488	64	0.04738	66	0.184	65.3
10to45	0.2669	67	0.2563	67	0.05153	68	0.192	67.3
15to40	0.2687	68	0.2621	69	0.04893	67	0.193	68.0
5to50	0.2695	69	0.2617	68	0.05248	69	0.195	68.7
10to40	0.2825	70	0.2716	71	0.05574	71	0.203	70.7

SEG

Age Trim	Simulations MRE	Simulations Rank	US Counties MRE	US Counties Rank	High-Income Countries MRE	High-Income Countries Rank	Average MRE	Average Rank
5to45	0.2844	71	0.2716	70	0.05885	72	0.205	71.0
15to35	0.2857	72	0.277	72	0.05399	70	0.206	71.3
10to35	0.2987	73	0.2847	73	0.06197	73	0.215	73.0
5to40	0.2993	74	0.2863	74	0.06559	74	0.217	74.0
5to35	0.3141	75	0.2926	75	0.0694	75	0.225	75.0
10to30	0.3147	76	0.2975	76	0.07101	76	0.228	76.0
5to30	0.329	77	0.3077	77	0.07851	77	0.238	77.0
5to25	0.3438	78	0.3263	78	0.0828	78	0.251	78.0

GGBSEG

Age Trim	Simulations MRE	Simulations Rank	US Counties MRE	US Counties Rank	High-Income Countries MRE	High-Income Countries Rank	Average MRE	Average Rank
50to70	0.0911	6	0.0917	5	0.03645	8	0.073	6.3
45to80	0.0926	7	0.0933	8	0.03588	5	0.074	6.7
45to70	0.0967	9	0.0911	4	0.03754	9	0.075	7.3
50to75	0.0888	5	0.0947	13	0.03562	4	0.073	7.3
45to75	0.0938	8	0.0934	9	0.03645	7	0.075	8.0
55to80	0.0846	2	0.0989	22	0.03251	1	0.072	8.3
55to75	0.0847	3	0.0975	20	0.03317	2	0.072	8.3
45to65	0.1008	12	0.0893	1	0.03963	13	0.077	8.7
50to80	0.0881	4	0.0965	16	0.03613	6	0.074	8.7
40to70	0.1029	13	0.091	3	0.03907	12	0.078	9.3
40to75	0.0996	11	0.0923	6	0.03811	11	0.077	9.3
40to80	0.0979	10	0.0937	10	0.03766	10	0.076	10.0
60to80	0.082	1	0.1013	28	0.03432	3	0.073	10.7
40to65	0.1073	16	0.0898	2	0.04085	15	0.079	11.0
35to75	0.1061	15	0.0931	7	0.04101	16	0.080	12.7
35to80	0.1038	14	0.0942	12	0.04056	14	0.080	13.3
35to70	0.11	17	0.0938	11	0.04257	20	0.082	16.0
30to70	0.1187	22	0.0961	14	0.0424	17	0.086	17.7
30to80	0.1111	18	0.0969	19	0.04249	18	0.084	18.3
30to75	0.1141	20	0.0965	15	0.0428	21	0.084	18.7
40to60	0.1131	19	0.0969	18	0.04256	19	0.084	18.7
35to65	0.1152	21	0.0968	17	0.04392	24	0.085	20.7
25to80	0.1201	23	0.1005	25	0.04283	22	0.088	23.3
35to60	0.1214	24	0.099	23	0.04325	23	0.088	23.3
30to65	0.1245	26	0.0985	21	0.04528	26	0.089	24.3
25to75	0.1239	25	0.1012	27	0.04414	25	0.090	25.7
35to55	0.1289	27	0.0995	24	0.04576	28	0.091	26.3
30to60	0.1315	30	0.1011	26	0.046	29	0.093	28.3
20to80	0.1305	29	0.1067	31	0.04557	27	0.094	29.0
25to70	0.1291	28	0.1048	29	0.04615	30	0.093	29.0
20to75	0.1348	31	0.1087	32	0.04897	32	0.097	31.7
30to55	0.1396	33	0.1051	30	0.04942	33	0.098	32.0
25to65	0.1355	32	0.1089	33	0.04823	31	0.098	32.0
15to80	0.1408	35	0.1116	36	0.05061	34	0.101	35.0
25to60	0.1433	36	0.1106	34	0.05134	35	0.102	35.0
20to70	0.1405	34	0.1121	37	0.05178	36	0.101	35.7
15to75	0.1458	37	0.1143	38	0.05283	37	0.104	37.3

GGBSEG

Age Trim	Simulations MRE	Simulations Rank	US Counties MRE	US Counties Rank	High-Income Countries MRE	High-Income Countries Rank	Average MRE	Average Rank
30to50	0.1492	39	0.1116	35	0.05292	38	0.105	37.3
20to65	0.1478	38	0.1156	40	0.05481	40	0.106	39.3
25to55	0.1525	42	0.1156	39	0.0546	39	0.108	40.0
15to70	0.1521	41	0.1158	41	0.05497	41	0.108	41.0
10to80	0.1513	40	0.116	42	0.05703	43	0.108	41.7
20to60	0.1563	43	0.1208	45	0.0556	42	0.111	43.3
10to75	0.1566	44	0.1188	43	0.05922	46	0.112	44.3
25to50	0.1629	46	0.1202	44	0.05889	44	0.114	44.7
15to65	0.1597	45	0.1225	46	0.05902	45	0.114	45.3
5to80	0.1649	48	0.1226	47	0.06101	47	0.116	47.3
20to55	0.1659	49	0.1244	48	0.06136	48	0.117	48.3
10to70	0.1635	47	0.1251	49	0.06301	52	0.117	49.3
5to75	0.171	51	0.1261	50	0.06149	49	0.120	50.0
15to60	0.1688	50	0.1279	51	0.06252	50	0.120	50.3
25to45	0.1748	53	0.1282	52	0.06298	51	0.122	52.0
10to65	0.1718	52	0.1311	53	0.06638	55	0.123	53.3
20to50	0.1769	54	0.1344	55	0.06532	53	0.126	54.0
5to70	0.1786	55	0.1332	54	0.06553	54	0.126	54.3
15to55	0.1792	56	0.1381	56	0.06749	56	0.128	56.0
10to60	0.1812	57	0.1414	58	0.06777	57	0.130	57.3
5to65	0.1875	58	0.1397	57	0.06983	60	0.132	58.3
20to45	0.19	59	0.1446	60	0.06822	58	0.134	59.0
15to50	0.1908	60	0.1445	59	0.07098	61	0.135	60.0
10to55	0.1918	61	0.1487	62	0.06948	59	0.137	60.7
5to60	0.1971	62	0.1468	61	0.07224	62	0.139	61.7
20to40	0.2047	65	0.1579	64	0.0724	63	0.145	64.0
10to50	0.2037	63	0.1587	65	0.0759	65	0.146	64.3
15to45	0.2041	64	0.161	66	0.07392	64	0.146	64.7
5to55	0.2081	66	0.1571	63	0.07677	66	0.147	65.0
15to40	0.2189	68	0.1678	68	0.07886	67	0.155	67.7
10to45	0.217	67	0.1686	69	0.0808	68	0.155	68.0
5to50	0.2203	69	0.1645	67	0.08484	69	0.157	68.3
10to40	0.2315	70	0.1752	71	0.09047	71	0.166	70.7

GGBSEG

	Simulations		US Counties		High-Income Countries		Average	
Age Trim	MRE	Rank	MRE	Rank	MRE	Rank	MRE	Rank
5to45	0.2341	71	0.1751	70	0.09202	72	0.167	71.0
15to35	0.2345	72	0.1787	72	0.08756	70	0.167	71.3
10to35	0.2466	73	0.185	73	0.09839	73	0.177	73.0
5to40	0.249	74	0.1866	74	0.09904	74	0.178	74.0
5to35	0.2647	76	0.2034	75	0.10833	75	0.192	75.3
10to30	0.2619	75	0.2101	76	0.11028	76	0.194	75.7
5to30	0.2806	77	0.2154	77	0.11491	77	0.204	77.0
5to25	0.296	78	0.2279	78	0.12469	78	0.216	78.0
