# Supplementary material for: What Can We Conclude from Death Registration? Improved Methods for Evaluating Completeness
Source: PLoS Med. 2010 Apr 13;7(4):e1000262. doi: 10.1371/journal.pmed.1000262 (PMC2854130; doi:10.1371/journal.pmed.1000262)
Supplement: Text S1 — Summary of DDMs. (0.07 MB PDF) [file pmed.1000262.s002.pdf]

Supporting Information Text S1: Summary of Death Distribution Methods

Generalized Growth Balance

The Generalized Growth Balance (GGB) method extends the Growth Balance Equation developed by Brass [1]. The original method employs the demographic relationship

$$\frac{N(a)}{N(a+)} = r + \frac{D(a+)}{N(a+)}$$

where $N(a)$ is the number of people turning age a in the interval under observation, $N(a+)$ is the total population above age a, r is the stable population growth rate, and $D(a+)$ is the total deaths at age a and over. Generalizing the equation to non-stable populations, i.e. populations with non-constant growth, Hill [2] estimated an r for each age group, yielding

$$\frac{N(a)}{N(a+)} = r(a+) + \frac{D(a+)}{N(a+)}$$

The method requires population by age at two points in time and the average annual deaths in between those points. If we have incomplete censuses and incomplete death registration, then we cannot observe $N1(a)$, the true population of people aged a at the first census, $N2(a)$, the true population of peopled aged a at the second census, or $D(a)$, the true number of deaths of people aged a in between the first and second censuses. We instead observe $N1^O(a)$, $N2^O(a)$, and $D^O(a)$, where

$$N1^O(a) = c_1 * N1$$

$$N2^O(a) = c_2 * N2$$

$$D^O(a) = v_1 * D(a)$$

In the above equations, c_1 is the completeness of the first census, c_2 is the completeness of the second census and v_1 is the completeness of death registration. Substituting these expressions into the Generalized Growth Balance equation allows us to estimate the relative completeness of the death registration to the censuses. After substitution and simplification, the equation becomes

$$\frac{N^O(a)}{N^O(a+)} = \frac{1}{t}\log\frac{c_1 N2^O(a+)}{c_2 N1^O(a+)} + \frac{\sqrt{c_1 c_2}}{v_1}\frac{D^O(a+)}{N^O(a+)}$$

By pulling the ratio $\frac{c_1}{c_2}$ out of the growth rate term and rearranging, we obtain

$$\frac{N^O(a)}{N^O(a+)} - r^O(a+) = \frac{1}{t}\log\frac{c_1}{c_2} + \frac{\sqrt{c_1 c_2}}{v_1}\frac{D^O(a+)}{N^O(a+)}$$

which defines a line with intercept $\frac{1}{t}\log\frac{c_1}{c_2}$ and slope $\frac{\sqrt{c_1 c_2}}{v_1}$. Least squares regression can estimate both the slope and intercept of this line and thus the relative completeness of the censuses and a correction factor for the mortality rates.

Synthetic Extinct Generations

The method of extinct generations, a precursor to the Synthetic Extinct Generations method (SEG), estimates the number of people aged a at time t, $N_t(a)$ by employing the following demographic relationship between $N_t(a)$ and $D_t(a)$, the number of deaths of people aged a at time t.

$$N_t(a) = D_t(a) + D_{t+1}(a+1) + D_{t+2}(a+2) + \cdots = \int_a^\infty D_{t+x-a}(x)dx$$

We can arrive at $N_t(a)$ either directly or indirectly by counting each person contributing to $N_t(a)$ when he or she dies [3]. The method of extinct generations is impractical for those who need the resulting death information now. Instead of waiting for cohorts to go extinct, SEG [4] estimates the number of future deaths in the cohort by adjusting D_t at all ages above a by a growth factor. The new approach yields the equation

$$N_t(a) = \int_a^{\infty} D_t(x) e^{\int_a^x r(u)du} dx,$$

where the right hand side of the equation represents the estimated deaths at older ages to the current cohort $N_t(a)$ by using current deaths at each age $D_t(x)$ from death registration systems and age specific growth rates $r(u)$ that can be obtained from comparing cohort sizes across two censuses. Dividing the estimated $N_t(a)$ calculated using the right hand side of the equation by the $N_t(a)$ observed directly from the census gives the relative completeness ratio $\frac{v_1}{c_1}$.

Generalized Growth Balance-Synthetic Extinct Generations

If the two censuses used to estimate the age-specific growth rates in SEG have different levels of completeness, then SEG's final estimate of the correction factor will be biased. The combined GGB-SEG method attempts to correct this bias. Hill and Choi [5] proposed multiplying the population numbers from the first census by the $\frac{c_2}{c_1}$ derived from the intercept of the Generalized Growth Balance equation. After this adjustment, the two censuses are complete with respect to each other and SEG will produce a less biased estimate of the correction factor.

References

1. Brass W (1996) Demographic data analysis in less developed countries: 1946-1996. Population Studies-A Journal of Demography 50: 451.

2. Hill K (1987). Estimating census and death registration completeness. Asian and Pacific Population Forum 1(3): 8-13.

3. Vincent P (1951). La mortalité des vieillards. Population 6: 182-204.

4. Bennett NG, Horiuchi S (1981). Estimating the completeness of death registration in a closed population. Population Index 47(2): 207-221.

5. Hill K, Choi Y (2004). Death distribution methods for estimating adult mortality: sensitivity analysis with simulated data errors. Paper prepared for Adult Mortality in Developing Countries Workshop. Marin County, CA: The Marconi Center, July 8-11.
